# Exhaled aerosols among PCR-confirmed SARS-CoV-2-infected children

**DOI:** 10.3389/fped.2023.1156366

**Published:** 2023-04-21

**Authors:** Pia Schuchmann, Gerhard Scheuch, Rolf Naumann, Marius Keute, Thomas Lücke, Stefan Zielen, Folke Brinkmann

**Affiliations:** ^1^ Department of Children and Adolescents, University Children’s Hospital, Ruhr University of Bochum, Bochum, Germany; ^2^ Pediatric Practice (Dr. Voigt, Dr. Heier), Stadtbergen, Germany; ^3^ GS Bio-Inhalation GmbH, Headquarters & Logistics, Gemuenden, Germany; ^4^ AspiAir GmbH, Gemuenden, Germany; ^5^ Independent Statistical Consultant, Warendorf, Germany; ^6^ Department for Children and Adolescents, Allergology, Pulmonology and Cystic Fibrosis, University Hospital, Goethe University, Frankfurt, Germany; ^7^ Department of Pediatric Pneumology, University Children's Hospital, Ruhr University of Bochum, Bochum, Germany; ^8^ Department of Pediatric Pneumology and Allergology, University Children’s Hospital Schleswig-Holstein, Lübeck, Germany

**Keywords:** aerosols, SARS-CoV-2 infection, COVID-19 vaccination, omicron, children

## Abstract

**Background:**

Available data on aerosol emissions among children and adolescents during spontaneous breathing are limited. Our aim was to gain insight into the role of children in the spread of severe acute respiratory syndrome coronavirus 2 (SARS-CoV-2) and whether aerosol measurements among children can be used to help detect so-called superspreaders—infected individuals with extremely high numbers of exhaled aerosol particles.

**Methods:**

In this prospective study, the aerosol concentrations of SARS-CoV-2 PCR-positive and SARS-CoV-2 PCR-negative children and adolescents (2–17 years) were investigated. All subjects were asked about their current health status and medical history. The exhaled aerosol particle counts of PCR-negative and PCR-positive subjects were measured using the Resp-Aer-Meter (Palas GmbH, Karlsruhe, Germany) and compared using linear regression.

**Results:**

A total of 250 children and adolescents were included in this study, 105 of whom were SARS-CoV-2 positive and 145 of whom were SARS-CoV-2 negative. The median age in both groups was 9 years (IQR 7–11 years). A total of 124 (49.6%) participants were female, and 126 (50.4%) participants were male. A total of 81.9% of the SARS-CoV-2-positive group had symptoms of viral infection. The median particle count of all individuals was 79.55 particles/liter (IQR 44.55–141.15). There was a tendency for older children to exhale more particles (1–5 years: 79.54 p/L; 6–11 years: 77.96 p/L; 12–17 years: 98.63 p/L). SARS-CoV-2 PCR status was not a bivariate predictor (*t* = 0.82, *p* = 0.415) of exhaled aerosol particle count; however, SARS-CoV-2 status was shown to be a significant predictor in a multiple regression model together with age, body mass index (BMI), COVID-19 vaccination, and past SARS-CoV-2 infection (*t* = 2.81, *p* = 0.005). COVID-19 vaccination status was a highly significant predictor of exhaled aerosol particles (*p* < .001).

**Conclusion:**

During SARS-CoV-2 infection, children and adolescents did not have elevated aerosol levels. In addition, no superspreaders were found.

## 1. Introduction

Severe acute respiratory syndrome coronavirus 2 (SARS-CoV-2) is a novel coronavirus that was first detected in Wuhan, China in December 2019 (1). SARS-CoV-2 is an enveloped RNA virus with a size of 60–160 nm, which is similar in size to influenza viruses (2, 3). In March 2020, the World Health Organization (WHO) declared the global spread of the pathogen a pandemic (4). By December 2022, over 640 million confirmed cases and over 6.6 million deaths attributed to the pandemic had been recorded (5).

Respiratory infections are transmitted through direct contact with an infected person, through indirect contact with a contaminated surface (fomite), or *via* droplets and aerosols in the surroundings of an infected person (6, 7). Interestingly, the airborne transmission of virus-laden particles (aerosol transmission) was not initially considered a relevant route of transmission (8, 9). Accordingly, the WHO declared on March 28, 2020, that the virus was transmitted *via* large droplets that fell to the ground close to infected individuals, as well as by touching contaminated surfaces (10). In addition, the WHO recommendation that masks help to control the transmission of SARS-CoV-2 was given rather late (11). Currently, it is well established that aerosols play a major role in SARS-CoV-2 transmission (9, 12). In particular, so-called “superspreading events”, in which a high number of SARS-CoV-2 infections occur, cannot be explained only by droplet transmission or smear infection (13, 14). The same holds true for the observed differences between indoor and outdoor transmission (15).

The physical behavior of exhaled aerosol particles depends on their size, density, and shape, as well as the ambient temperature, humidity, and air circulation, among other factors (8). During normal breathing, the human lungs produce aerosol particles. These particles are generated in the peripheral airways during inhalation by the reopening of collapsed airways and are released during exhalation (16–19). Studies have already shown that there is interindividual variation in aerosol emissions as well as variation between different activities (20–22). For instance, it has been shown that aerosol production increases during singing or shouting (22–25). Most respiratory aerosol particles are much smaller than 5 µm and therefore can penetrate deep into the respiratory tract to the bronchioli and alveoli (26–30). Approximately 85% of all exhaled SARS-CoV-2 RNA was found in particles smaller than 5 µm (31).

Children infected with coronavirus disease 2019 (COVID-19) often present with mild or no symptoms, and life-threatening conditions or death are rare (32). This might be explained by the lower prevalence of preexisting conditions such as hypertension, diabetes, and pulmonary disease among children or by higher exposure to other seasonal coronaviruses, leading to higher antibody titers in children, which might give them some protection against SARS-CoV-2 (33–35). Moreover, it is known that the binding of the SARS-CoV-2 spike protein to angiotensin-converting enzyme-2 (ACE2) allows the virus to enter cells (36). It has been hypothesized that the function of ACE2 is not yet mature in children, and therefore, its binding capacity is lower (34, 37). Bunyavanich et al. suggested that lower expression of ACE2 in the nasal epithelium of children results in lower susceptibility to SARS-CoV-2 infection (38). The role of ACE2 is still under debate, however, and more research is needed (36).

Unlike among adults, there is little knowledge about aerosol emissions among children (39). The question of whether children contribute substantially to the spread of SARS-CoV-2 has not yet been resolved, despite many observational studies being conducted. The aim of this prospective study was to gain insights into aerosol emission among children and adolescents. Specifically, we aimed to gain further insight into the role of children in SARS-CoV-2 transmission and to examine the differences in aerosol concentrations and particle size between SARS-CoV-2 PCR-positive and SARS-CoV-2 PCR-negative children and adolescents.

## 2. Methods

### 2.1. Study design

A monocentric prospective study was conducted to investigate the concentrations and size distribution of exhaled aerosols from SARS-CoV-2-positive and SARS-CoV-2-negative children and adolescents aged 2–17 years. PCR analysis for SARS-CoV-2 was performed in all subjects who did not have a current SARS-CoV-2 PCR test result (max. interval 48 h) at the time of measurement. The study was approved by the Ethics Committee of the Medical Faculty of the Ruhr-University Bochum (number 21-7365) and registered under DRKS00028539 in the German Register of Clinical Studies (DRKS). Detailed information was provided to the subjects and their guardians, and the aim and procedure of the study, as well as potential risks, were explained. Written informed consent was obtained from all study participants.

The study was supported by the Palas Company which provided the measurement equipment and by the “Ina und Gerhard Scheuch Stiftung für Aerosolforschung”.

### 2.2. Subject recruitment and data collection

The subjects were recruited between November 2021 and April 2022. Potential study participants or their guardians were recruited at cooperating pediatricians' offices, schools, and sport clubs, either by approaching them directly or by handing them written information. Parents received further written information after expressing interest.

Participants in the study were between 2 and 17 years of age and were divided into three age groups (2–5, 6–11, 12–17 years). Aerosol measurements were performed in subjects with and without acute SARS-CoV-2 infection. Acute respiratory infection was defined by the presence of at least one of the following symptoms: cough, rhinitis, and fever, with symptom onset in the previous 72 h. Healthy subjects were SARS-CoV-2 negative as well as free of signs of acute upper respiratory tract infection, such as rhinitis, cough, or fever.

Subjects who were unable to undergo the aerosol measurement, understand the content of the study, or did not have consent provided by their guardian were excluded from the study.

Due to the course of the current SARS-CoV-2 pandemic, longitudinal measurement of subjects was not feasible.

### 2.3. Clinical investigations

Before aerosol measurement, all subjects or their guardians answered questions regarding their current health status (weight, height), previous diseases (especially cardiac and pulmonary), allergies, medication, coronavirus vaccination status, past SARS-CoV-2 infection, physical fitness and tobacco exposure. Subjects with acute upper respiratory tract infection were asked about the time course and symptoms (cough, rhinitis, fever, etc.). All subjects were tested for SARS-CoV-2 *via* PCR before aerosol measurement. Variant-specific PCR tests were used to detect virus variants (e.g., Delta or Omicron) in most of the participants.

### 2.4. Aerosol measurement

Measurements of the exhaled particle concentrations and size distribution were performed using the Resp-Aer-Meter (Palas GmbH, Karlsruhe, Germany). For this purpose, the principle of optical light scattering by means of a white light LED sensor of the Fidas® system was used. With this measuring method, exhaled particles between 145 nm and 10 µm could be detected with high resolution. In the aerosol sensor, a polychromatic light source creates a precisely defined optical measurement volume. Scattered light pulses are generated by the particles when they pass the optical light source. The particle number and size are determined by the number and intensity of the scattered light pulses. The temperature and relative humidity in the exhaled air were also measured and considered. An integrated heating element prevented condensation and allowed larger droplets to evaporate before they reached the sensor (40).

**Figure 1:**
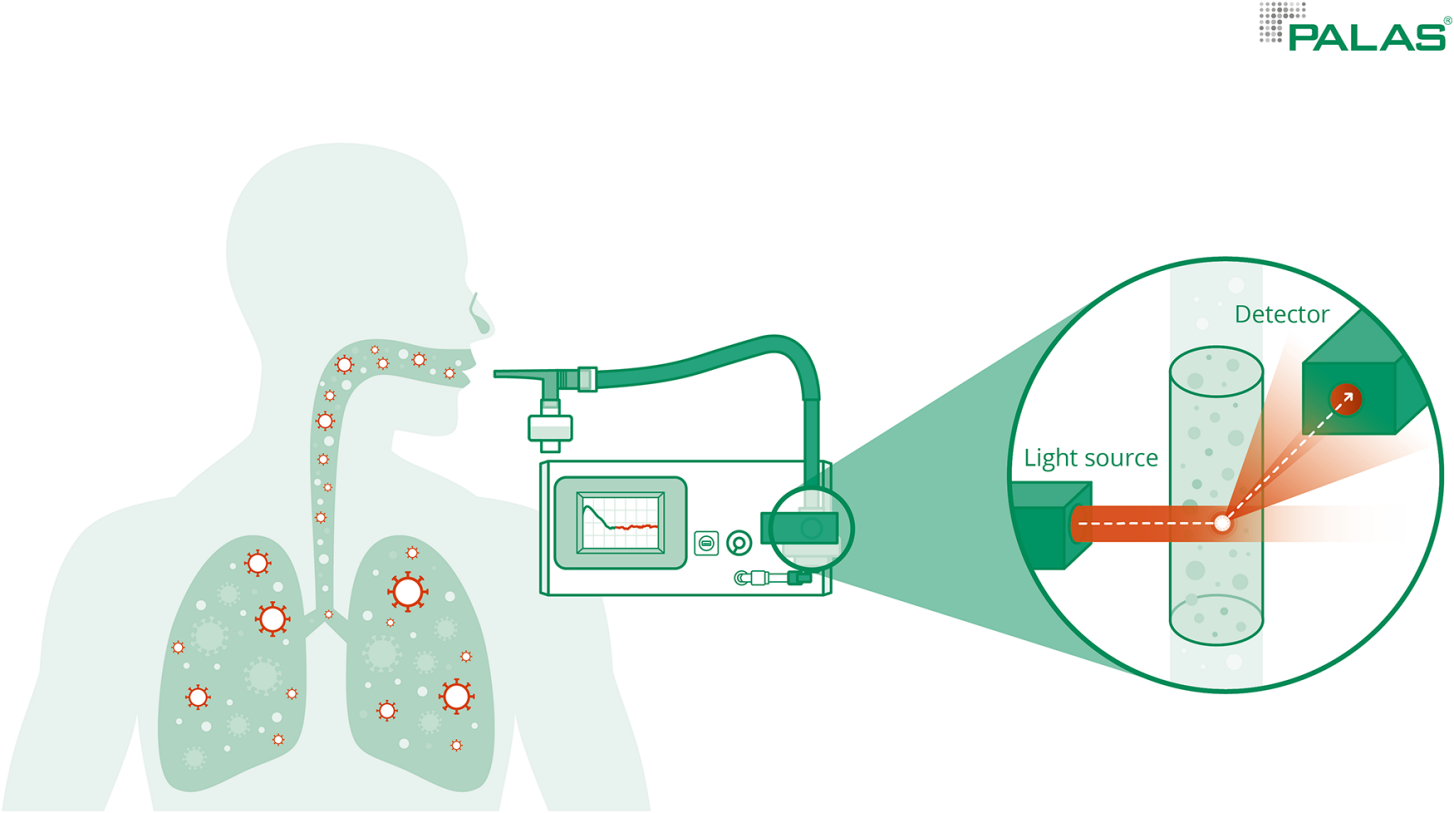
Measurement of aerosols. Measurement of the exhaled particle concentrations and size distribution was performed using the Resp-Aer-Meter using the principle of optical light scattering.

During the measurement, subjects inhaled and exhaled orally through a mouthpiece. The mouthpiece was connected to a HEPA filter *via* a T-piece which was connected to the Resp-Aer-Meter *via* a tube. Particle-free air was inhaled through the HEPA filter. Nasal breathing was prevented during the measurement process by a nose clip so that the measurement would not be influenced by environmental aerosols. Air was continuously drawn in from the Resp-Aer-Meter *via* a T-piece for sampling and directed to the sensor. A sterile-packed breathing set was used for each measurement. The breathing set consisted of a mouthpiece, T-piece, antistatic connecting tubing, HEPA filter, and nose clip (Figure 1).

**Figure 2:**
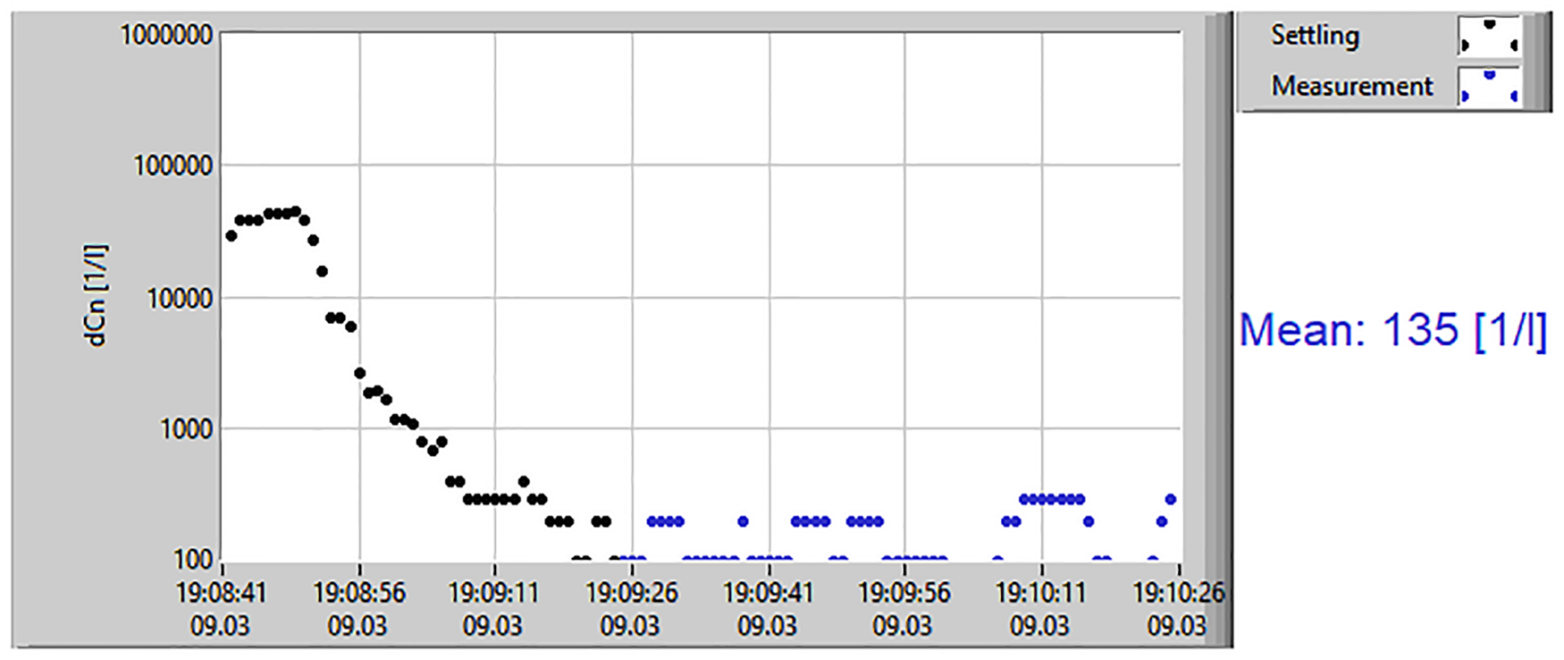
Results of aerosol measurement. The measurement results display the mean value of exhaled particles/liter, including a graphical chart of the measurement course.

Prior to each measurement, a leak test was performed to ensure that the breathing set was fixed to the Resp-Aer-Meter without leakage. During the first minute of breathing, the washout phase took place. During this phase, environmental aerosol particles that were present in the lungs were washed out. This caused a rapid decrease in the particle concentration in the exhaled air. After a few breaths, the concentration did not further decline, and only the particles produced in the lungs were measured. The subsequent measurement phase lasted approximately 60–90 s. The results of the measurement were immediately available for documentation and evaluation. In addition to the mean value of the exhaled particle count/liter, the direct evaluation included a graphical chart of the measurement course and the aerosol particle size distribution (Figure 2).

### 2.5. Endpoints

The primary endpoint of this study was the aerosol concentration in exhaled air measured in particles/liter in children and adolescents with and without SARS-CoV-2 infection.

The secondary endpoints of the study were the influence of confounders such as age, sex, height, weight, BMI, symptoms, tobacco exposure, COVID-19 vaccination status, and SARS-CoV-2 infection on the exhaled aerosol concentration. In addition, the particle size distribution was analyzed.

### 2.6. Statistical methods

Statistical analysis was performed with R 4.1. The main hypothesis tested was that a current positive SARS-CoV-2 infection status was predictive of the particle count in the breath aerosol. Several additional demographic and health-related predictors, including age, sex, allergies, previous infections and diseases, and vaccination status, were considered.

All statistical testing was carried out using linear regression models, with the log-transformed particle count as the target variable. First, bivariate regression models were fitted for each predictor separately. Subsequently, all predictors were entered into a common regression model together with the SARS-CoV-2 infection status to detect potential interaction patterns.

Finally, a subset of predictors that applied only to SARS-CoV-2-positive patients, such as the time since symptom onset, or that were otherwise correlated with SARS-CoV-2 infection status, such as respiratory symptoms, were discarded. The remaining predictors were entered into a common multiple linear regression model, which included the main effects and interaction with the SARS-CoV-2 infection status for each predictor. This model was subjected to an automatized model selection procedure using the MASS::stepAIC function in R, which identified the subset of the original parameters that minimized the model's value on the Akaike Information Criterion (AIC). The AIC is a deviation measure for models that is penalized by model complexity (number of parameters). Hereafter, this model is referred to as the optimized model/regression.

## 3. Results

A total of 250 children and adolescents were included in this study. Of these subjects, 105 subjects tested positive for SARS-CoV-2 by PCR, and 145 subjects tested negative. Interestingly, testing for SARS-CoV-2 variants was performed in 89 (85%) subjects, and all tests (100%) were positive for the omicron variant. The characteristics of the subjects are shown in Table 1. The mean age of the SARS-CoV-2-positive group was slightly higher than that of the SARS-CoV-2-negative group (11 vs. 8 years). The sex distribution was almost balanced in both groups, with a slight predominance of the male sex in the SARS-CoV-2-positive group (54.3% vs. 47.6%). The median BMI was 17.96 in the SARS-CoV-2-positive group and 16.46 in the SARS-CoV-2-negative group.

**Table 1:** Characteristics of severe acute respiratory syndrome coronavirus 2 (SARS-CoV-2) polymerase chain reaction (PCR)-positive and PCR-negative children and adolescents.

	SARS-CoV-2 PCR positive (*n* = 105)	SARS-CoV-2 PCR negative (*n* = 145)	Total (*n* = 250)
**Sex**			
Female	48 (45.7%)	76 (52.4%)	124 (49.6%)
Male	57 (54.3%)	69 (47.6%)	126 (50.4%)
**Age (years)**			
Median	11	8	9
Range	4–17	2–17	2–17
**BMI (kg/m^2^)**			
Median	17.96	16.46	16.91
Range	13.32–38.51	11.89–27.76	11.89–38.51
**Comorbidities**			
Allergy	19 (18.1%)	19 (13.1%)	38 (15.2%)
Coughing	60 (57.1%)	25 (17.2%)	85 (34%)
Rhinitis	65 (61.9%)	39 (26.9%)	104 (41.6%)
Fever	28 (26.7%)	1 (0.7%)	29 (11.6%)
Sore throat (*n* = 104)	25 (46.3%)	1 (2%)	26 (25%)
COVID-19 vaccination	39 (37.1%)	20 (13.8%)	59 (23.6%)
Past SARS-CoV-2 infection	10 (9.5%)	25 (17.2%)	35 (14%)
Smoke exposure	31 (29.5%)	30 (20.7%)	61 (24.4%)

### 3.1. Medical history

Preexisting medical conditions were present in only 4.8% (5/105) of subjects in the SARS-CoV-2-positive group and in 2.8% (4/145) of the SARS-CoV-2-negative group. Allergies were reported in 18.1% (19/105) of SARS-CoV-2-positive subjects and in 13.1% (19/145) of SARS-CoV-2-negative subjects. A total of 9.5% (10/105) of SARS-CoV-2-positive subjects compared to 17.3% (25/145) of SARS-CoV-2-negative subjects were previously infected with SARS-CoV-2.

In the SARS-CoV-2-positive group, 37.1% (39/105) compared to 13.8% (20/145) of the SARS-CoV-2-negative subjects had been vaccinated for COVID-19. In the SARS-CoV-2-positive group, 29.5% of subjects lived in a smoking household; in the SARS-CoV-2-negative group, 20.87% of subjects lived in a smoking household.

As expected, 81.9% (86/105) of subjects in the SARS-CoV-2-positive group had symptoms of viral infection. The following symptoms were reported: cough in 57.1% (60/105) of subjects, rhinitis in 61.9% (65/105) of subjects, sore throat in 46.3% (25/54) of subjects, and fever in 26.7% (28/105) of subjects. In the SARS-CoV-2-negative group, 29% (42/145) of subjects had symptoms of acute infection and cough was reported in 17.2 (25/245) of subjects, rhinitis was reported in 26.9% (39/145) of subjects, sore throat was reported in 2% (1/50) of subjects, and fever was reported in 0.7% (1/145) of subjects.

### 3.2. Aerosol measurements

**Figure 3:**
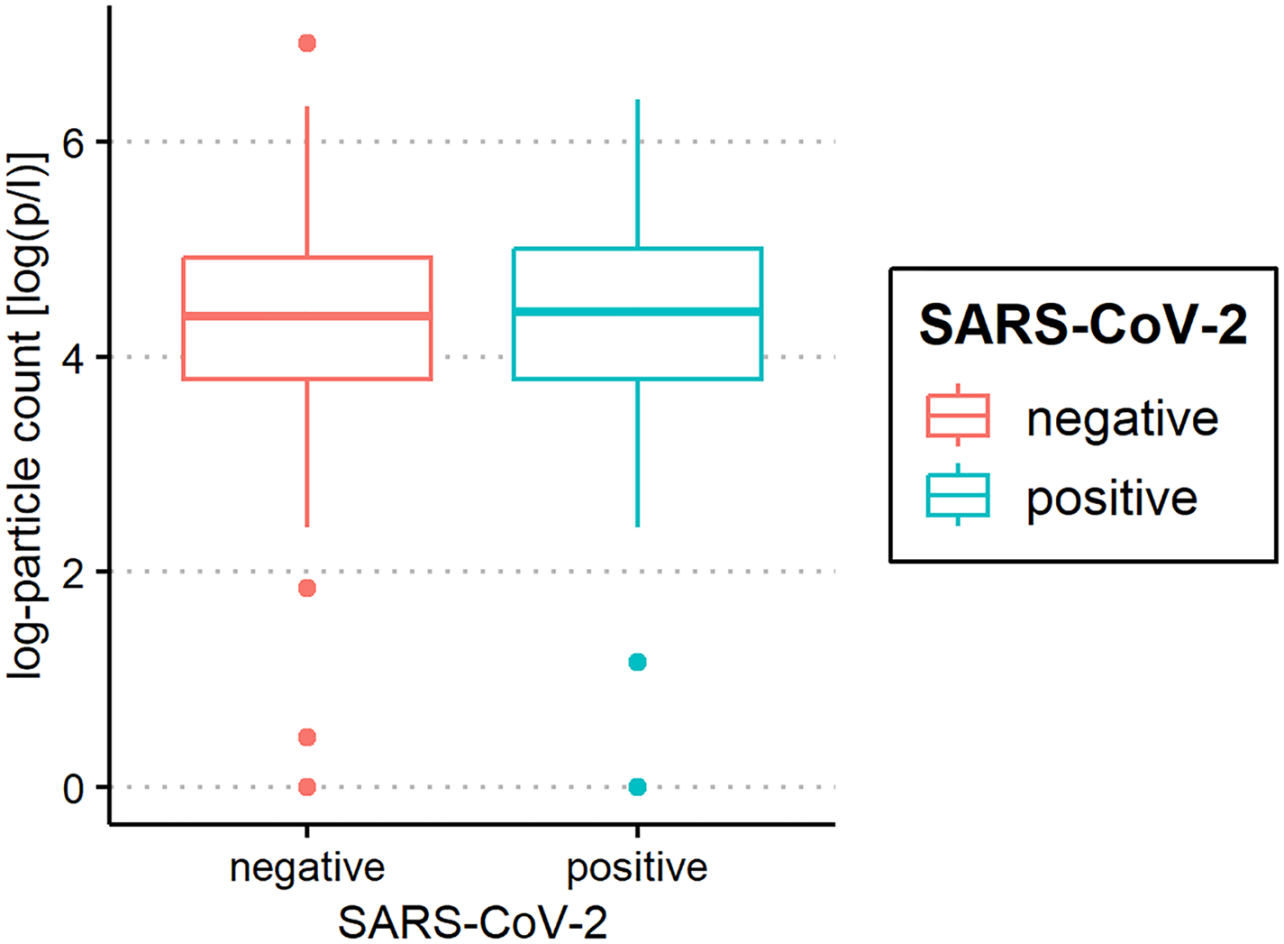
Aerosol measurement of severe acute respiratory syndrome coronavirus 2 (SARS-CoV-2) polymerase chain reaction (PCR) -positive and PCR-negative children and adolescents. Exhaled particle counts (in particles/liter, displayed on a logarithmic scale, *y*-axis) in SARS-CoV-2- PCR-positive and PCR-negative children and adolescents (*x*-axis).

**Figure 4:**
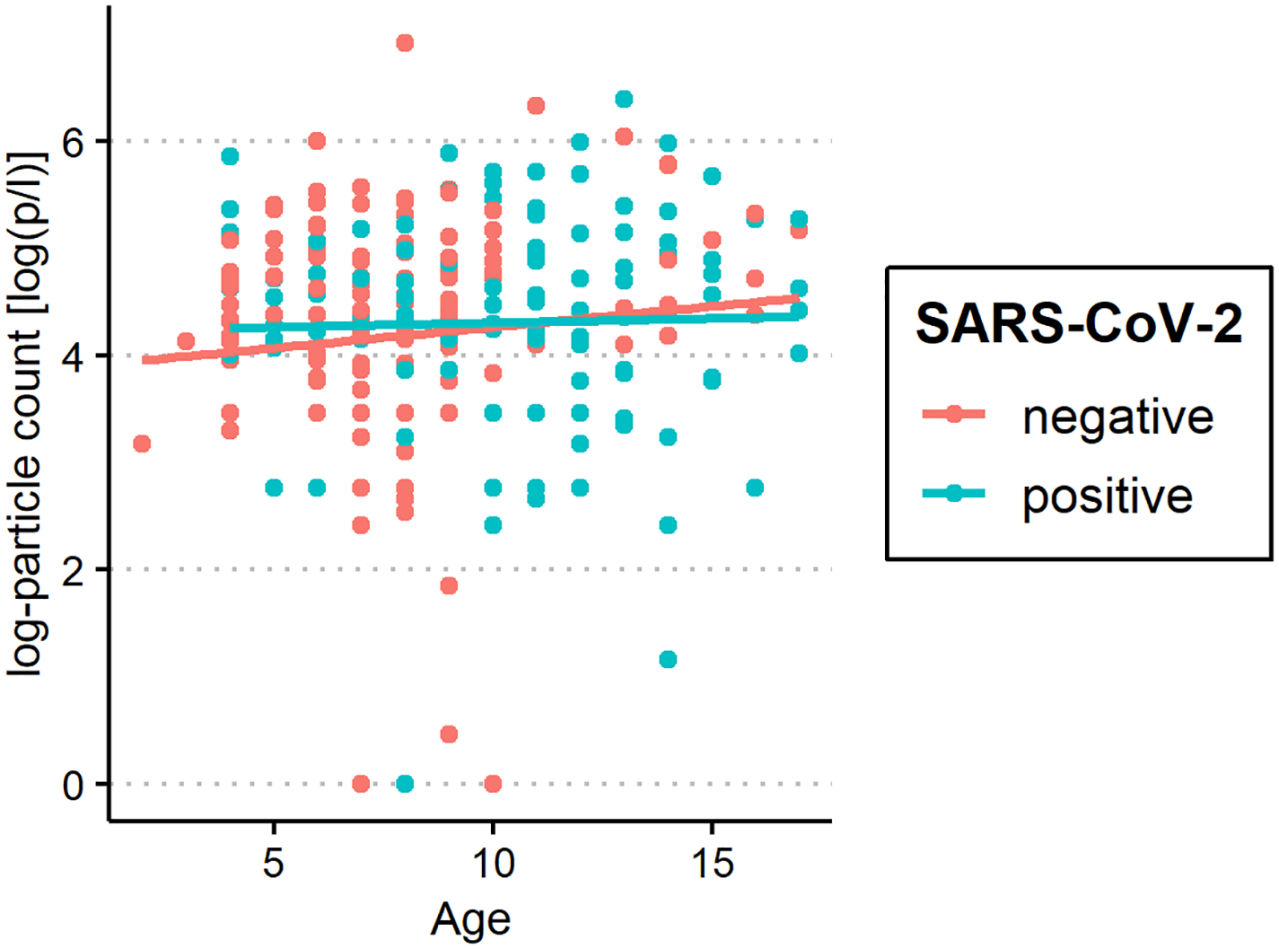
Aerosol measurement in relation to age among severe acute respiratory syndrome coronavirus 2 (SARS-CoV-2) polymerase chain reaction (PCR)-positive and PCR-negative subjects. Exhaled particle counts (in particles/liter, displayed on a logarithmic scale, *y*-axis) in SARS-CoV-2 PCR-positive and PCR-negative children and adolescents displaying the relation to age (*x*-axis).

**Figure 5:**
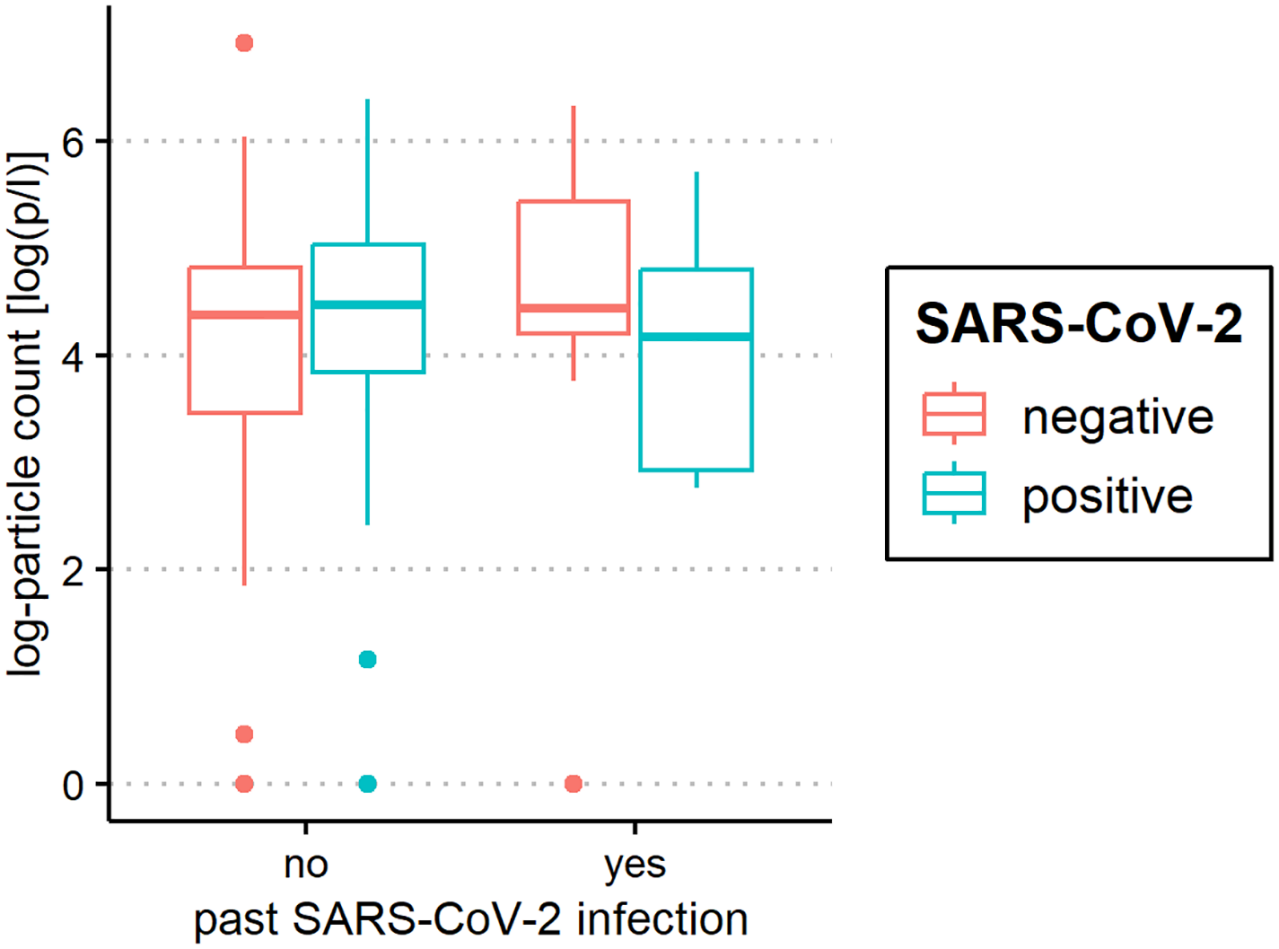
Aerosol measurement in relation to past severe acute respiratory syndrome coronavirus 2 (SARS-CoV-2) infection. Exhaled particle counts (in particles/liter, displayed on a logarithmic scale, *y*-axis) in SARS-CoV-2 polymerase chain reaction (PCR) -positive and PCR-negative children and adolescents displaying the relation to past coronavirus disease 2019 (COVID-19) infection (*x*-axis).

**Figure 6:**
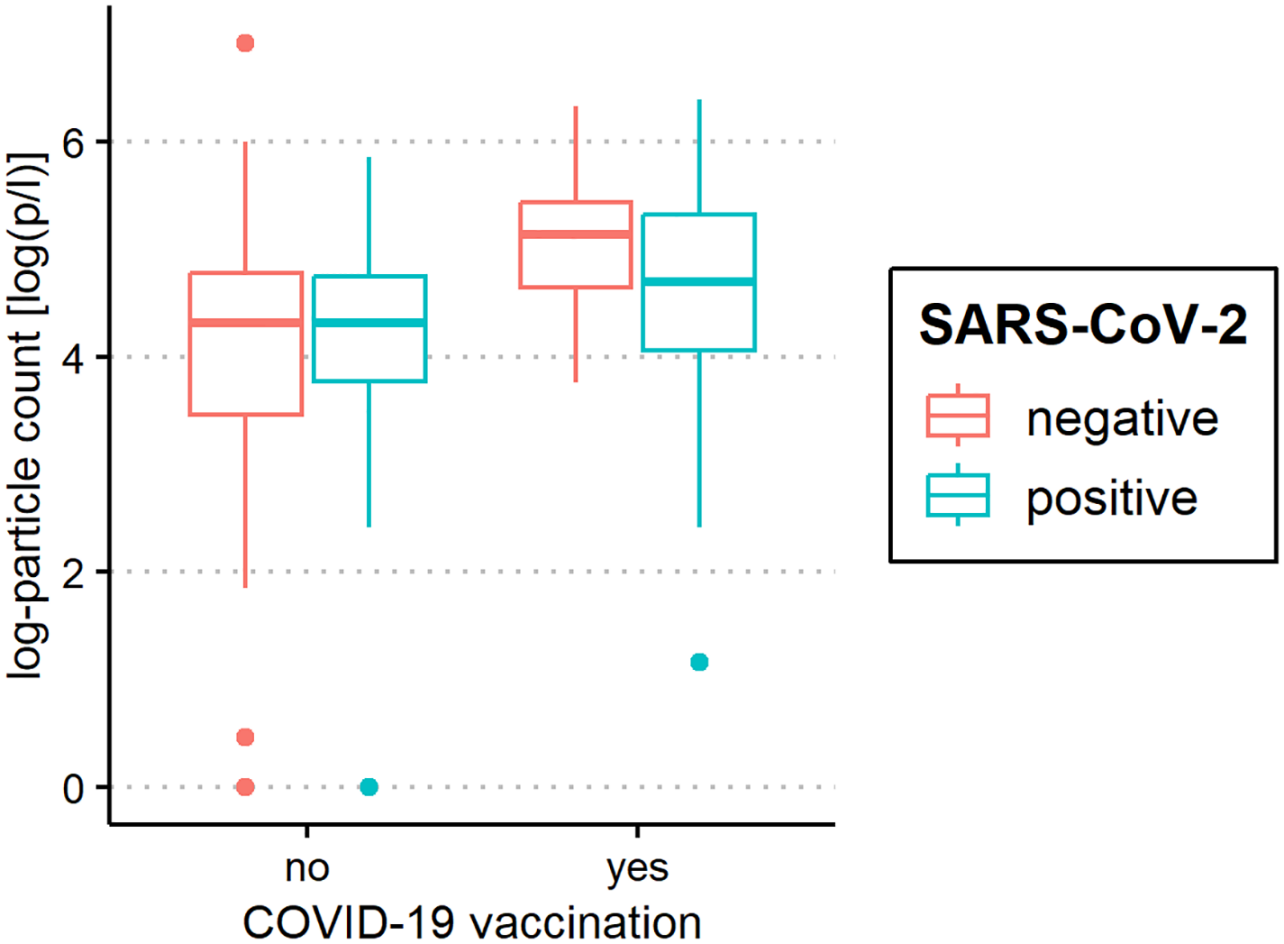
Aerosol measurement in relation to coronavirus disease 2019 (COVID-19) vaccination status. Exhaled particle counts (in particles/liter, displayed on a logarithmic scale, *y*-axis) in severe acute respiratory syndrome coronavirus 2 (SARS-CoV-2) polymerase chain reaction (PCR) -positive and PCR-negative children and adolescents displaying the relation to COVID-19 vaccination status (*x*-axis).

**Figure 7:**
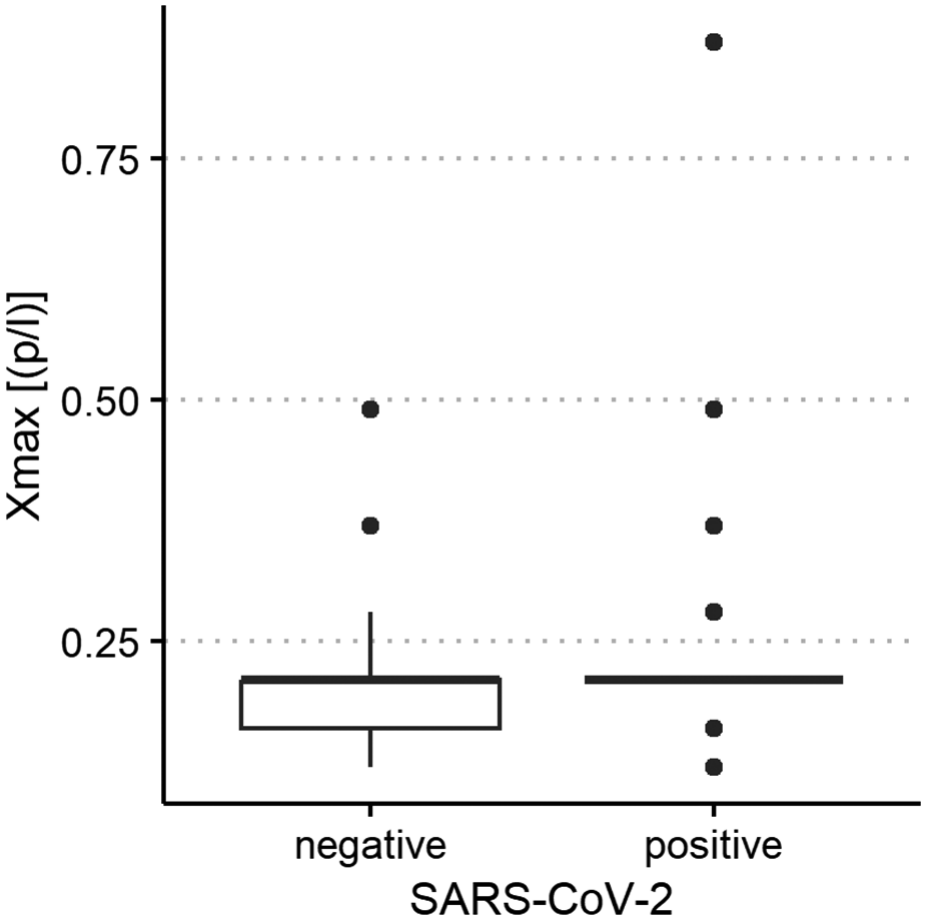
Aerosol particle size distribution of severe acute respiratory syndrome coronavirus 2 (SARS-CoV-2) polymerase chain reaction (PCR)-positive and PCR-negative children and adolescents. Maximum size of exhaled aerosol particles (in µm, displayed on the *y*-axis) in SARS-CoV-2 PCR-positive and PCR-negative children and adolescents (*x*-axis).

The median of all subjects' (SARS-CoV-2-positive and SARS-CoV-2-negative) mean particle counts was 79.55 p/L (IQR 44.55–141.15). Between SARS-CoV-2-positive subjects (median 82.72 p/L, IQR 44.55–149.52) and SARS-CoV-2-negative subjects (median 79.55, IQR 44.55–136.78), there was no significant difference in the number of exhaled particles (Figure 3). In the bivariate regression model, the aerosol particle count did not differ between the positive and negative groups (*t* = 0.82, *p* = 0.415). However, SARS-CoV-2 status was shown to be a significant predictor in the optimized model when controlling for the influence of age, BMI, vaccination status and past SARS-CoV-2 infection (*t* = .2.81 *p* = 0.005). Age had no effect on the aerosol particle count (bivariate model: *t* = 1.18, *p* = 0.24, optimized model: *t* = −1.68, *p* = 0.094), but there was a tendency for adolescents (age group 12–17) to exhale more particles than younger children (age group 2–5; 98 vs. 79 p/L) (Figure 4). Past SARS-CoV-2 infection was not a bivariate predictor (*t* = 1.4, *p* = 0.163) of the number of aerosol particles, but in the common regression model with age, BMI, SARS-CoV-2 status, and vaccination status, it showed a significant association with the number of exhaled aerosol particles (*t* = 2.26, *p* = 0.025) (Figure 5). The median mean particle count was 133 p/L among subjects with prior COVID-19 vaccination vs. 74.8 p/L among subjects without COVID-19 vaccination. Therefore, COVID-19 vaccination status was a highly significant predictor of exhaled aerosol particle count in the bivariate as well as the optimized model (*p* < 0.001) (Figure 6). In addition, there were no significant differences in the number of exhaled aerosol particles due to sex (*p* = 0.263), cough (*p* = 0.934), rhinitis (*p* = 0.472), sore throat (*p* = 0.423), fever (*p* = 0.343), allergies (*p* = 0.31), tobacco exposure (*p* = 0.332), and preexisting conditions (*p* = 0.605). There was a significant difference in the size distribution of exhaled aerosols between SARS-CoV-2- positive and SARS-CoV-2-negative subjects (*p* = 0.041). Although the median particle size in both groups was 0.21 µm, the distribution was much narrower in the SARS-CoV-2-positive group. The particle size was predominantly less than 0.5 µm in both groups (Figure 7).

## 4. Discussion

The primary route of transmission of SARS-CoV-2 is *via* aerosols, as previously demonstrated in several studies (12, 30, 41). Children and adolescents have been shown to transmit the virus, yet they do not appear to be the drivers of virus spread (13, 41). It has been reported several times that transmission from adults to children is more common than vice versa (37, 42). Overall, children and adolescents appear to be less infectious than adults with SARS-CoV-2 (33, 43, 44). Presumably, the lower expression of ACE-2 receptors in children also results in lower susceptibility to SARS-CoV-2 since SARS-CoV-2 enters the body *via* human ACE-2 receptors (37, 38).

We found particle concentrations in children to be substantially lower than those in healthy adults, where an average concentration of 252 p/L has been reported previously using the same method (39). This might be due to the lower number of alveoli and terminal bronchioli in children's respiratory tracts, as these structures are thought to be the origin of aerosol production (41, 45, 46). Additionally, differences in respiratory maneuvers as well as surfactant production may influence aerosol production. Children also have a lower respiratory minute volume than adults. Edwards et al. described a correlation between the number of aerosol particles exhaled and age (47). However, a direct relationship between aerosol particle exhalation and age group was not observed in this study, but there was a tendency for older children to exhale more particles than younger children (98 vs. 79 p/L).

Contrary to previous findings of Edwards et al. and Gutmann et al. in adults, children suffering from SARS-CoV-2 infection were not found to exhale significantly more aerosol particles than uninfected children in this study (82.72 vs. 79.55 p/L). Additionally, no infected children were found to breathe more than 595 p/L, whereas so-called superspreaders in studies among adults exhaled >5,000 particles/liter and accounted for 15.6% of infected adults (39). At first glance, our aerosol measurements do not match the data from Gutmann et al. (48). This study reported significantly increased exhaled aerosol levels in SARS-CoV-2 PCR-positive children and adolescents (median 355.0 p/L) compared to SARS-CoV-2 PCR-negative participants (median 195.0 p/L; *p* < 0.001). One possible explanation is that Gutmann et al. measured aerosols when the delta variant was most prevalent from February–December 2021, whereas most of our measurements were performed when the omicron variant was the predominant variant. Indeed, 85% of our SARS-CoV-2-PCR-positive subjects were tested for virus variants, and in all cases, the omicron variant was detected, which is the predominant virus currently in Germany. Although the symptoms, such as runny nose, fever, and cough, of the delta and omicron COVID-19 variants are similar, several studies have shown that omicron causes milder disease. Apparently, patients infected with the omicron variant had less involvement of the lower respiratory tract and a reduced likelihood of hospital admission (49–52). There is increasing evidence that the SARS-CoV-2 omicron variant exhibits altered cell tropism to escape the immune pressure against ACE2-dependent viral entry provided by vaccination (53). Interestingly, it was demonstrated that the omicron variant replicates faster than other SARS-CoV-2 variants studied in the bronchi but less efficiently in the lung parenchyma (54). Thus, it is tempting to speculate that the delta variant may induce higher levels of aerosols due to higher parenchyma involvement than the omicron variant (55). In addition, Gutman et al. showed that patients with respiratory failure and pneumonia had significantly higher aerosol levels than patients with mild COVID-19 infection in ambulatory care (39).

Interestingly, in this study, the number of particles increased significantly after COVID-19 vaccination. The underlying mechanism of this finding is unclear. There are some reports that surfactant production is altered during COVID-19 infection (56, 57). Sinnberg et al. demonstrated that IgA autoantibodies to pulmonary surfactant proteins B and C are detectable in patients with COVID-19 and that these autoantibodies impair the ability of pulmonary surfactant to decrease surface tension (56). However, we do not know if such a phenomenon is present after COVID-19 vaccination.

This study had some limitations. For example, only mildly ill, nonhospitalized participants were included. It seems reasonable to assume that the number of exhaled aerosol particles is higher, especially in severely ill SARS-CoV-2-positive individuals, for whom respiratory tract damage can be assumed. Additionally, the measurement could not always be performed at the same time as the PCR test, but there was a maximum of 48 h between the measurement of the number of aerosol particles and the PCR test. The participants were not tested for any other respiratory pathogens besides SARS-CoV-2. Measurements of SARS-CoV-2-positive subjects were performed at different time points during the infection. Longitudinal measurements would be needed to assess the dynamics of aerosol production during the course of infection. Different environmental factors could also have influenced the results. Ambient conditions such as the humidity, season (weather, pollen count, etc.), environmental aerosols (urban area vs. rural area), were not held constant, even though they may play a role in the formation as well as in the measurement of aerosol particles. In addition to the interindividual variation in the measurement of exhaled aerosol particles, the measurements were also influenced by the participant's compliance and breathing technique. In particular, it was difficult for some young children of preschool and primary school age to keep the mouthpiece completely tight during the measurement and carry out the study procedures until completion. In this study, the majority of subjects were infected with the omicron variant of SARS-CoV-2. Previous studies that were carried out earlier in the pandemic likely had a very different distribution of SARS-CoV-2 variants, which might have influenced the number of exhaled aerosol particles. In this study, PCR or viral cultures of virus-containing particles were not analyzed. Furthermore, the participants were not asked to provide the exact date of their vaccination, so there are no data available about the time elapsed between vaccination and measurements.

In summary, the values of exhaled aerosol particles in children and adolescents are substantially lower overall than those in the studies conducted thus far in adults. Our results indicate that the measurement of exhaled aerosol particles is not suitable as a testing tool in children and adolescents to interrupt chains of infection. Although this study has shown that aerosol measurements in children and adolescents cannot detect and break chains of infection, it has provided further insight into aerosol production in this age group. Further studies are needed to gain a better understanding of the influencing factors on aerosol production.

## Data Availability

The raw data supporting the conclusions of this article will be made available by the authors, without undue reservation.
